# The association between dietary polyphenol intake and cardiometabolic factors in overweight and obese women: a cross-sectional study

**DOI:** 10.1186/s12902-022-01025-3

**Published:** 2022-05-10

**Authors:** Yasaman Aali, Sara Ebrahimi, Farideh Shiraseb, Khadijeh Mirzaei

**Affiliations:** 1 Department of Community Nutrition, School of Nutritional Sciences and Dietetics, Tehran University of Medical Sciences (TUMS), P.O. Box: 14155-6117, Tehran, Iran; 2 Institute for Physical Activity and Nutrition, School of Exercise and Nutrition Sciences, Deakin University, Geelong, VIC 3220 Australia

**Keywords:** Flavonoid, Phenolic acid, Stilbenes, Lignan, Obesity, Cardiovascular disease, Diabetes mellitus

## Abstract

**Objective:**

The previous evidence shows that there is an association between total dietary polyphenols intake (DPI) and its subclasses and lower risk of metabolic Syndrome (MetS). This cross-sectional study aims to evaluate associations between DPI and cardiometabolic factors in Iranian women.

**Methods:**

A total of 404 Iranian women were included in this study. Dietary intakes and polyphenols intakes were measured using a validated semi-quantitative food frequency questionnaire (FFQ) and the Phenol-Explorer database, respectively. Biochemical variables and blood pressure were evaluated using Pars Azmoon kits and mercury sphygmomanometer.

**Results:**

The mean intake of total polyphenol was 2533.96 ± 1223.67 g. While there were significant negative associations between stilbenes and lignans intake and body mass index (BMI) (*P*-value = 0.04; *P*-value = 0.02, respectively), beverages containing phenolic acids and hip circumference (HC) (*P*-value = 0.02), total polyphenols intake and weight to hip ratio (WHR) (*P*-value = 0.04). Also there was significant negative associations between stilbenes intake and cholesterol (CHOL) level (*P*-value = 0.03), other polyphenols intake and triglyceride (TG) ((*P*-value = 0.01), lignan intake and homeostasis model assessment insulin resistance (HOMA-IR) (*P*-value = 0.03).

**Conclusion:**

These findings demonstrated that dietary polyphenols were associated with cardiometabolic factors in Iranian women. Prospective and interventional studies in both genders, different populations and ethnicities need to be conducted to further the knowledge about examine associations between consumption of polyphenols and metabolic component.

**Supplementary Information:**

The online version contains supplementary material available at 10.1186/s12902-022-01025-3.

## Introduction

Metabolic Syndrome (MetS) is a chronic disease with a low-grade inflammatory condition [1, 2]. While the worldwide prevalence of MetS in adults is 25%, the prevalence of MetS was 36% among Iranian adults in 2016, with the higher prevalence among women than in men [3–5]. MetS is identified through different criteria including abdominal obesity (WC: male: $\ge$ 102, female: $\ge$ 88 cm), insulin resistance, hypertension (SBP: $\ge$ 130, DBP: $\ge$ 85 mm/Hg), higher level of triglycerides (TG: $\ge$ 150 mg/dl) and low-density lipoprotein cholesterol levels (LDL-c), and lower level of high-density lipoprotein cholesterol (HDL-c) [2]. Given, people with Mets are more probable to develop chronic diseases including diabetes mellitus (DM), cardiovascular disease (CVD), and atherosclerosis, examining the related aspects of MetS is of significance [6, 7]. While Mets impose a major economic burden on the society [6, 7], it could be addressed through modifying the lifestyle and improving dietary intakes [8].

It has been demonstrated that an unhealthy diet, alongside other behavioral risk factors could play a main role in developing non communicable chronic diseases including obesity, and diabetes [9]. Previous evidence showed that diets with lower intake of antioxidants are associated with more probability of developing MetS [10]. Polyphenols are the main components of many plants which could be found in various foods and beverages including fruits, vegetables, tea, and the evidence showed that polyphenols may be effective in reducing the components of MetS including the risk of type 2 diabetes and CVD [11, 12]. Polyphenol compounds have been reported to have different subgroups including flavonoids, stilbenes, phenolic acids, and lignans [13]. A 6-year cohort study showed that higher intake of certain classes of dietary polyphenols including flavanones and lignans were associated with lower body mass index (BMI) and waist circumference (WC) in adults[14]. Furthermore, a study on Polish women showed that there were significant associations between high dietary polyphenols intake (DPI) and lower fasting plasma glucose (FPG), blood pressure, and HDL-c [15, 16].

Given the higher prevalence of MetS in women and considering that there is no study that examined associations between DPI and its components and metabolic outcomes in overweight and obese Iranian women, this study aimed to examine associations between DPI and cardiometabolic factors such as body composition and biochemical parameters in overweight and obese women.

## Materials and methods

### Subjects and study design

This cross sectional study was conducted on overweight and obese Iranian women who referred to the healthcare centers in Tehran, Iran. Multistage random sampling was used to recruit 404 women aged range between 18 to 48 years old according to inclusion and exclusion criteria. In the present study, BMI > 25 kg/m^2^ was considered as an index of obesity and overweight in participants. The inclusion criteria included consent to participate in the study, the health condition of participants, female gender, BMI between 25–40 kg/m^2^. The exclusion criteria included pregnancy, lactation, menopause, use of lipid and blood sugar lowering or weight loss tablets, alcohol consumption, smoking, history of high blood pressure, diabetes, renal, liver, cardiovascular, thyroid and cancer disease, adherence to weight loss diets and any particular dietary regimen in the last 6 months [17, 18]. Participants with implausible daily energy intakes higher than 4200 kcal/d or lower than 800 kcal/d were also excluded [19]. The study was approved by the ethical committee at Tehran University of Medical Sciences and informed written consent was obtained from each participant. All procedures involving human subjects were approved by the (IR.TUMS.MEDICINE.REC.1399.637) and all methods were carried out in accordance with relevant guidelines. The research was funded by the Tehran University of Medical Sciences (Grant number: 98–3-212–46,721).

### Dietary assessment

Dietary data was collected by trained dietitian, using a semi-quantitative questionnaire with 147 food items listed that its validity and reliability has been confirmed by previous studies [20, 21]. Individuals reported their frequency of consumption of a given serving of each food item during the previous year on a daily, weekly, monthly, or yearly basis. Portion sizes of the consumed foods were converted to grams using household measurements [22] and dietary data was analyzed using the Nutrition IV software (First Data Bank, San Bruno, CA).

### Calculation of DPI

Data on the total polyphenol content in foods (Supplementary table 1) were measured from the Phenol-Explorer database (www. phenol explorer.eu/contents) [23]. The total polyphenol content was measured using the Folin Ciocalteu assay or sum of four main subgroups (including flavonoids, phenolic acids, stilbenes, lignans, and other polyphenols).

### Socio-demographic characteristics, lifestyle characteristics, and other covariates

Sociodemographic characteristics including age, education, occupation, marital status, and medical history including consumption of medications and supplements were collected by a trained nutritionist. Physical activity was measured using the International Physical Activity Questionnaire (IPAQ) that its validity and reliability have been confirmed in the previous study that was conducted by Craig et al. 2003 [24]. This questionnaire consists of seven questions. Each question consists of two sections (number of movements per week and duration), and each section indicates participant ‘s level of physical activity (vigorous, moderate, walking, and inactive).

### Anthropometric measurements

Anthropometric indices were measured by trained dietitian. Weight was measured with an accuracy of 100 g using a Seca scale (made in Germany) with the least clothes and without shoes. Height was measured with an accuracy of 0.5 cm using a Seca scale without shoes. Waist circumference (WC) was measured in the narrowest area of the waist by a non-elastic tape with an accuracy of 0.5 cm. Neck circumference (NC) and hip circumference (HC) were measured with the accuracy of 1 mm and 0.5 cm, respectively. BMI was calculated (weight (kg) / height (m^2^)) for every participants. Furthermore, the waist-to-hip ratio (WHR) (WC (cm)/ hip (cm)) and the waist to height ratio (WHtR) (WC (cm)/ height (cm)) were calculated.

### Biochemical assessments

Participants were referred to the Nutrition and Biochemistry Laboratory of the school of Nutritional and Dietetics at Tehran University of medical sciences. Venous blood samples were collected after 10–12 h overnight fasting. The EDTA anticoagulant plasma and serum samples were separated after centrifuging for 15 min at 3000 rpm, and the remaining blood was washed three times with 0.9% NaCl solution. After serum separation, it was frozen immediately at -80 °C for laboratory assessments. Serum concentrations of TG, total cholesterol (CHOL), HDL-c, LDL-c, glucose, and insulin were measured using Pars Azmoon laboratory kits (test Pars Inc, Tehran, Iran). Levels of TG, total CHOL, HDL-c, LDL-c, and fasting blood glucose (FBG) were measured using glycerol-3-phosphate oxidase phenol 4-amino antipyrine peroxidase (GPO-PAP), enzymatic endpoint, direct enzymatic clearance, and glucose oxidase phenol 4-amino, respectively. Insulin resistance (IR) (mIU/ml) was measured through the homeostatic model assessment (HOMA). HOMA-IR was calculated according to the following equation: [FPG (mmol/l) × fasting plasma insulin (µIU/l)] / 22.5 [25]. Systolic and diastolic blood pressure (SBP, DBP) were measured three times using a mercury sphygmomanometer and after resting for 15 min using a mercury.

### Statistical analyses

Statistical analysis was conducted using the IBM SPSS software version 25.0 (SPSS, Chicago, IL, USA) and *P*-value < 0.05 was considered statistically significant. Variables were reported as means and standard deviations (SD) for continuous variables and categorical variables as number and percentage. The Kolmogorov–Smirnov test was used to determine the normal distribution of variables. For classification categories of DPI was used visuals binning. A one-way analysis of variance (ANOVA) test was used to analyses continuous variables and Chi-square test was used to compare qualitative variables according to tertiles of DPI (less than 1984.59 (first tertile), between 1984.59–2731.68 (secondary tertile) and more than 2731.68 (third tertile)). Linear regression analysis was used to examine associations between DPI and MetS and its components. Analysis of covariance (ANCOVA) test was used to adjust the analysis for confounders including age, BMI, physical activity, intake of energy, and supplement. Findings were reported as Beta (B) and 95% confidence intervals (CIs). Post-Hoc (Bonferroni) analyses, were masured to detect a significant mean difference of variables across tertiles of DPI.

## Result

### Study population characteristics

A total number of 404 women were included in the analysis, and baseline characteristics are given in Table 1. The mean intake of energy, total polyphenol, flavonoids, phenolic acids, other polyphenols, lignans and stilbenes was 2633.809.43 kcal, 2533.96 ± 1223.67 g, 107.81 ± 58.32 g, 67.95 ± 43.11 g, 119.17 ± 59.58 g, 0.04 ± 0.05 g, and 3.52 ± 1.91 g, respectively. Other characteristics of the participants are reported in Table 1. In the present study, obese or overweight women according to the criteria of the metabolic syndrome had only a higher WC and were normal in terms of other criteria such as biochemical parameters.

**Table 1:** Baseline characteristics of participants

**Variables**	Mean	SD
Age (year)	36.67	9.10
Weight (kg)	80.28	11.05
BMI (kg/m^2^)	30.98	3.90
WC (cm)	99.16	9.42
WHR (cm)	1.16	4.54
WHtR (cm)	0.61	0.05
FBG (mg/dl)	87.49	9.64
CHOL(mg/dl)	185.30	35.77
TG (mg/dl)	118.10	58.88
HDL-c (mg/dl)	46.58	10.86
LDL-c (mg/dl)	95.30	24.12

All data are presented as mean and SD

*BMI* body mass index, *WC* waist circumference, *HC* hip circumference, *NC* neck circumference, *WHR* weight to hip ratio, *WHtR* weight to height ratio, *FBG* fasting blood glucose, *CHOL* cholesterol, *TG* triglyceride, *HDL-c* high-density lipoprotein, *LDL-c* low-density lipoprotein, *HOMA* homeostasis model assessment, *IR* insulin resistance, *Hs-CRP* high-sensitivity C-reactive protein

### Association between characteristics of study population across tertiles of DPI (energy-adjusted)

After adjusting for potential confounders (age, physical activity, BMI, intake of energy, and supplement), there were significant mean differences for WHR (*P* = 0.04), HOMA IR (*P* = 0.05), SBP (*P* = 0.05) and LDL-c (*P* = 0.04) over among tertiles of DPI (Table 2).

**Table 2:** Characteristics of study population according to tertiles of DPI in overweight and obese women (*n* = 404)

**Characteristics**	**Tertile of DPI**			***p*-value**	*p*-value*
	**T1** **(< **1984.59**)**	**T2** **(**1984.59–2731.68**)**	**T3** **(> **2731.68**)**		
	**Mean** $\pm$ **SD**	**Mean** $\pm$ **SD**	**Mean** $\pm$ **SD**		
**Body composition**					
** BMI (kg/m**^**2**^**)**	31.54 ± 4.20	30.74 ± 3.83	30.61 ± 3.51	0.11	0.38
** WC (cm)**	101.11 ± 9.07	97.82 ± 9.60^a^	98.37 ± 9.29^a^	**0.01**	0.07
** HC (cm)**	106.05 ± 11.42	105.29 ± 5.72	105.58 ± 6.05	0.75	0.45
** NC (cm)**	37.60 ± 4.29	38.50 ± 12.26	36.80 ± 2.29	0.37	0.43
** WHR (cm)**	1.64 ± 7.98	0.93 ± 0.05	0.93 ± 0.05	0.35	**0.04**
** WHtR (cm)**	0.62 ± 0.05	0.60 ± 0.05	0.61 ± 0.05	0.09	0.04
**Biochemical parameters**					
** FBG (mg/dl)**	86.09 ± 9.21	89.53 ± 10.95	86.76 ± 8.50	**0.06**	0.26
** CHOL (mg/dl)**	184.18 ± 36.58	185.39 ± 39.66	185.58 ± 33.38	0.96	0.98
** TG (mg/dl)**	114.79 ± 51.59	117.91 ± 60.50	120.90 ± 64.43	0.81	0.53
** HDL-c (mg/dl)**	45.81 ± 9.93	47.28 ± 11.86	47.07 ± 10.62	0.68	0.57
** LDL-c (mg/dl)**	93.55 ± 23.79	94.09 ± 26.29	96.75 ± 22.77	0.65	**0.04**
** Insulin (mIU/ ml)**	1.19 ± 0.21	1.24 ± 0.23	1.20 ± 0.23	0.26	0.18
** HOMA IR**	3.50 ± 1.50	3.47 ± 1.36	3.13 ± 1.03	0.11	**0.05**
** SBP (mmHg)**	111.32 ± 13.59	112.53 ± 13.18^a^	111.22 ± 14.32^a^	0.76	**0.05**
** DBP (mmHg)**	78.44 ± 9.69	77.68 ± 11.69	76.75 ± 9.85	0.54	**0.06**

The use of $\mathrm{chi}-\mathrm{square}$, t-test, and ANOVA test

All data are presented as mean, SD or N and %

The analysis was adjusted for energy intake

*BMI* body mass index, *WC* waist circumference, *HC* hip circumference, *NC* neck circumference, *WHR* weight to hip ratio, *WHtR* weight to height ratio, *FBG* fasting blood glucose, *CHOL* cholesterol, *TG* triglyceride, *HDL-c* high-density lipoprotein, *LDL-c* low-density lipoprotein, *HOMA* homeostasis model assessment, *IR* insulin resistance, *Hs-CRP* high-sensitivity C-reactive protein, *SBP* systolic blood pressure, *DBP* diastolic blood pressure

^a^significant compared to tertile 1

*P*-value < 0.05 was considered significant

*P*-value * obtained from ANCOVA test adjusted for age, physical activity, BMI, total energy intake, and supplement consumption

### Daily food and nutrient intake according to tertiles of DPI (energy-adjusted)

After adjusting for confounders including (age, physical activity, BMI, total energy intake, and supplement consumption), there was a significant positive association between DPI and intake of whole grains, vegetables, fruits, legume (*P* < 0.001), sugar-sweetened beverages (*P* = 0.03), starchy vegetables (*P* = 0.001), spices (*P* = 0.02), carbohydrate (*P* = 0.03), total fat (*P* = 0.04), total fiber, vitamin B9, vitamin C, vitamin K, potassium, lutein, copper (*P* < 0.001), vitamin B6 and retinol (*P* = 0.001), lycopene (*P* = 0.03) and biotin (*P* = 0.002), as well as marginal significant differences for vitamins B1 and B12 (*P* = 0.06) (Supplementary table 2).

### Association between total polyphenol intake (energy-adjusted) and body composition and biochemical parameters

After adjusting for potential confounders (age, physical activity, intake of energy and supplement), there was a significant negative association between total polyphenol intake and WHR (β:—6.29; CI: 0.001, 0.002), and a marginal negative significant association between total polyphenol intake and WHtR (β:—6.72; CI: 0.001, 0.003) (Table 3).

**Table 3:** Association between DPI and its subtypes with health outcomes in overweight and obese women (*n* = 404)

Characteristics Model		Total polyphenols (mg/day)			Flavonoids (mg/day)					Phenolic acids (mg/day)			
		**B**	**CI**	**R**^**2**^	***P*-value**	**B**	**CI**	**R**^**2**^	***P*-value**	**B**	**CI**	**R**^**2**^	***P*-value**
**Body composition**													
**BMI (kg/m**^**2**^**)**	**Crude**	0.00	-0.00,0.00	0.007	0.27	0.00	-0.00,0.01	0.006	0.34	-0.00	-0.02,0.07	0.009	0.31
	**Adjusted**	0.00	-0.00,0.00	0.56	0.14	0.00	-0.01,0.01	0.04	0.66	-0.00	-0.01,0.01	0.04	0.85
**WC (cm)**	**Crude**	-0.00	-0.00,0.00	0.007	0.13	0.01	-0.01,0.04	0.01	0.30	-0.01	-0.05,0.01	0.005	0.28
	**Adjusted**	-0.00	-0.00,0.00	0.23	**0.03**	0.00	-0.03.0.03	0.004	0.98	0.00	-0.03,0.04	0.03	0.89
**HC (cm)**	**Crude**	-7.97	-0.00,0.00	0.000	0.83	0.00	-0.02,0.02	0.003	0.69	-0.00	-0.03,0.02	0.006	0.58
	**Adjusted**	0.00	-0.00,0.00	0.06	0.18	0.00	-0.02,0.02	0.07	0.91	0.00	-0.02,0.02	0.08	0.76
**NC (cm)**	**Crude**	-0.00	-0.00,0.00	0.09	0.20	0.01	-0.01,0.04	0.001	0.38	0.00	-0.03,0.03	0.001	0.98
	**Adjusted**	-0.00	-0.00,-0.00	0.23	**0.04**	0.01	-0.01,0.04	0.02	0.36	-0.00	-0.04,0.03	0.02	0.82
**WHR (cm)**	**Crude**	0.00	-0.01,0.001	0.002	0.35	-0.00	-0.01,0.01	0.00	0.87	0.00	-0.01,0.01	0.000	0.77
	**Adjusted**	-6.29	0.001,0.002	0.25	**0.04**	5.59	0.00,0.00	0.03	0.95	-2.41	0.00,0.00	0.03	0.82
**WHtR (cm)**	**Crude**	-3.67	-0.001,0.001	0.01	0.17	0.00	0.00,0.00	0.009	0.23	0.00	0.00,0.00	0.01	0.12
	**Adjusted**	-6.72	0.001,0.003	0.25	**0.05**	4.24	0.00,0.00	0.04	0.96	-9.26	0.00,0.00	0.03	0.93
**Biochemical parameters**													
**FBG(mg/dl)**	**Crude**	0.00	-0.00,0.00	0.004	0.44	-0.00	-0.03.0.03	0.004	0.96	0.00	-0.03,0.04	0.000	0.81
	**Adjusted**	-0.01	-0.00,-0.00	0.13	0.07	-0.00	-0.04,0.03	0.00	0.81	0.00	-0.03,0.05	0.13	0.67
**CHOL (mg/dl)**	**Crude**	0.00	-0.00,0.00	0.003	0.12	0.00	-0.12,0.12	0.003	0.98	-0.10	-0.24,0.04	0.006	0.15
	**Adjusted**	-0.00	-0.00,-0.00	0.13	**0.04**	-0.00	-0.14,0.12	0.12	0.90	-0.06	-0.20,0.08	0.12	0.39
**TG (mg/dl)**	**Crude**	-0.00	-0.00,0.00	0.000	0.67	0.13	-0.07,0.33	0.002	0.21	-0.11	-0.35,0.12	0.01	0.33
	**Adjusted**	0.00	-0.00,0.00	0.11	0.92	0.11	-0.13,0.35	0.12	0.38	-0.07	-0.34,0.19	0.11	0.56
**HDL-c(mg/dl)**	**Crude**	0.00	-0.00,0.00	0.001	0.46	0.03	-0.07,0.00	0.04	0.09	0.03	-0.01,0.07	0.000	0.14
	**Adjusted**	0.00	-0.00,0.00	0.01	0.09	0.02	-0.06,0.00	0.02	0.07	0.01	-0.02,0.06	0.01	0.44
**LDL-c (mg/dl)**	**Crude**	0.00	-0.00,0.00	0.002	0.39	-0.01	-0.10,0.06	0.03	0.66	-0.00	-0.10,0.09	0.00	0.87
	**Adjusted**	-0.00	-0.00,-0.00	0.15	**0.05**	0.00	-0.09,0.09	0.13	0.98	-0.02	-0.12,0.08	0.13	0.69
**Insulin (mIU/ ml)**	**Crude**	5.51	0.00,0.00	0.001	0.66	0.00	0.00,0.00	0.00	0.43	0.00	-0.00,0.00	0.000	0.48
	**Adjusted**	1.17	0.00,0.00	0.13	0.43	0.00	0.00,0.00	0.13	0.30	-0.00	-0.00,0.00	0.12	**0.06**
**HOMA IR**	**Crude**	0.00	-0.00,0.01	0.01	0.36	0.00	-0.00,0.00	0.002	0.28	-0.00	-0.00,0.00	0.003	0.52
	**Adjusted**	-0.00	-0.00,-0.00	0.16	**0.06**	0.00	-0.00.00	0.24	0.28	-0.00	-0.00,0.00	0.15	0.29
**SBP (mmHg)**	**Crude**	0.00	-0.00,0.00	0.00	0.74	0.01	-0.03,0.06	0.000	0.49	-0.03	-0.09,0.01	0.002	0.19
	**Adjusted**	-0.00	-0.00,0.00	0.10	0.50	0.01	-0.03,0.06	0.09	0.54	-0.03	-0.09,0.02	0.10	0.27
**DBP (mmHg)**	**Crude**	0.00	-0.00,0.00	0.009	0.40	0.01	-0.02,0.04	0.005	0.40	-0.03	-0.07,0.01	0.01	0.11
	**Adjusted**	0.10	-0.03,0.25	0.08	0.14	0.99	-23.85,25.85	0.26	0.93	-0.04	-0.09,0.001	0.28	**0.02**

Characteristics Model		Lignans (mg/day)				Stilbenes (mg/day)				Other polyphenols(mg/day)			
		**B**	**CI**	**R**^**2**^	***P*-value**	**B**	**CI**	**R**^**2**^	***P*-value**	**B**	**CI**	**R**^**2**^	***P*-value**
**Body composition**													
**BMI (kg/m**^**2**^**)**	**Crude**	-1.10	-9.44,7.24	0.001	0.79	-0.20	-0.46,0.04	0.01	0.10	0.00	-0.00,0.00	0.00	0.50
	**Adjusted**	-1.85	-12.10,-0.40	0.14	**0.02**	-0.34	-0.77,-0.09	0.16	**0.04**	0.00	-0.00,0.01	0.04	0.22
**WC (cm)**	**Crude**	4.25	-15.83,24.35	0.03	0.67	-0.62	-1.23,-0.01	0.008	**0.04**	0.00	-0.01,0.02	0.001	0.55
	**Adjusted**	2.42	-22.27,27.12	0.03	0.84	-0.70	-1.76,-0.05	0.16	**0.06**	0.01	-0.00,0.04	0.04	0.15
**HC (cm)**	**Crude**	-3.17	-20.83,14.49	0.009	0.72	-0.29	-0.82,0.24	0.001	0.28	0.00	-0.01,0.01	0.005	0.62
	**Adjusted**	2.52	-13.82,18.87	0.09	0.78	-0.51	-1.21,0.18	0.06	**0.04**	0.00	-0.01,0.02	0.07	0.46
**NC (cm)**	**Crude**	-2.98	-23.27,17.30	0.000	0.77	-0.47	-1.16,0.21	0.01	0.17	0.00	-0.01,0.02	0.002	0.54
	**Adjusted**	-5.19	-28.75,18.36	0.02	0.66	-0.59	-1.60,0.02	0.000	0.07	0.01	-0.01,0.03	0.02	0.40
**WHR (cm)**	**Crude**	2.15	-7.54,11.85	0.000	0.66	0.01	-0.28,0.31	0.000	0.93	0.00	-0.00,0.00	0.000	0.97
	**Adjusted**	0.04	-0.09,0.17	0.03	0.54	-0.00	-0.00,0.00	0.14	0.49	8.87	0.00,0.00	0.03	0.21
**WHtR (cm)**	**Crude**	0.00	-0.12,0.12	0.001	0.99	-0.00	-0.00,0.00	0.01	**0.03**	3.72	0.00,0.00	0.000	0.47
	**Adjusted**	-0.01	-0.16,0.13	0.03	0.86	-0.00	-0.01,-0.00	0.05	**0.04**	0.00	0.00,0.00	0.03	0.15
**Biochemical parameters**													
**FBG(mg/dl)**	**Crude**	-0.74	-24.83,23.34	0.001	0.95	-0.31	-1.05,0.42	0.006	0.39	-0.01	-0.03,0.01	0.02	0.36
	**Adjusted**	-25.26	-53.57,-0.05	0.14	**0.05**	-0.13	-1.28,1.01	0.13	0.82	0.01	-0.01,0.04	0.13	0.41
**CHOL (mg/dl)**	**Crude**	31.60	-59.34,122.54	0.001	0.49	-0.31	-3.09,2.47	0.001	0.82	-0.02	-0.11,0.05	0.000	0.52
	**Adjusted**	16.30	-78.74,111.36	0.12	0.73	-1.53	-5.38,-2.32	0.12	**0.03**	0.02	-0.07,0.11	0.12	0.67
**TG (mg/dl)**	**Crude**	29.62	-118.85,178.10	0.01	0.69	-0.84	-5.40,3.70	0.000	0.71	-0.17	-0.04,-0.11	0.04	**0.01**
	**Adjusted**	11.82	-167.75,191.41	0.14	0.89	-1.88	-9.20,5.44	0.11	0.61	-0.23	-0.05,0.41	0.18	**0.01**
**HDL-c(mg/dl)**	**Crude**	-7.36	-34.20,19.46	0.001	0.58	-0.48	-1.31,0.33	0.02	0.24	0.00	-0.02,0.02	0.000	0.93
	**Adjusted**	-23.49	-52.88,5.88	0.02	0.11	-0.47	-1.67,0.71	0.02	0.42	0.01	-0.01,0.04	0.01	0.37
**LDL-c (mg/dl)**	**Crude**	-1.36	-62.54,59.81	0.000	0.96	-0.58	-2.45,1.29	0.004	0.54	0.00	-0.06,0.05	0.000	0.86
	**Adjusted**	6.86	-60.14,73.89	0.13	0.84	-0.88	-3.60,1.83	0.13	0.52	0.00	-0.06,0.07	0.13	0.93
**Insulin (mIU/ ml)**	**Crude**	0.20	-0.37,0.78	0.002	0.48	-0.01	-0.03,0.00	0.004	0.18	0.00	-0.00,0.00	0.000	0.62
	**Adjusted**	0.19	-0.49,0.89	0.13	0.57	-0.01	-0.03,-0.01	0.13	0.07	0.00	-0.00,0.00	0.13	0.49
**HOMA IR**	**Crude**	-0.75	-4.10,2.59	0.007	0.65	-0.06	-0.16,0.03	0.007	0.18	-0.00	-0.00,0.00	0.007	0.32
	**Adjusted**	-2.22	-6.19,-1.73	0.25	**0.03**	-0.03	-0.19,0.00	0.14	0.07	0.00	-0.00,0.00	0.000	0.68
**SBP (mmHg)**	**Crude**	-0.27	-32.53,31.80	0.001	0.98	-0.20	-1.15,0.73	0.000	0.66	0.01	-0.01,0.04	0.02	0.35
	**Adjusted**	-3.45	-39.62,0.72	0.19	**0.06**	0.39	-1.15,1.94	0.09	0.61	0.02	-0.01,0.06	0.10	0.15
**DBP (mmHg)**	**Crude**	9.34	-14.67,33.36	0.000	0.44	0.35	-1.06,0.35	0.004	0.32	-0.01	-0.03,0.01	0.001	0.32
	**Adjusted**	0.42	-0.63,1.49	0.05	0.42	-0.00	-0.00,0.00	0.05	**0.05**	0.00	-0.02,0.02	0.05	0.78

All data are presented as B ± CI obtained from a linear regression

The analysis was adjusted for energy intake

*BMI* body mass index, *WC* waist circumference, *HC* hip circumference, *NC* neck circumference, *WHR* weight to hip ratio, *WHtR* weight to height ratio, *FBG* fasting blood glucose, *CHOL* cholesterol, *TG* triglyceride, *HDL-c* high-density lipoprotein, *LDL-c* low-density lipoprotein, *HOMA* homeostasis model assessment, *IR* insulin resistance,, *SBP* systolic blood pressure, *DBP* diastolic blood pressure

*P*-value for adjustment model: Adjusted for age, physical activity intake of energy and supplement

*P* –value < 0.05 was considered significant

### Association between phenolic acids intake (energy-adjusted) and body composition and biochemical parameters

After adjusting for potential confounders, there was a significant negative association between phenolic acids intake and DBP (β:—0.04; CI:—0.09, -0.001) (Table 3).

### Association between lignan intake (energy-adjusted) and body composition and biochemical parameters

After adjusting for potential confounders, there was a significant negative association between lignan intake and BMI (β:—1.85; CI:—12.10,—0.40), HOMA IR (β:—2.22; CI:—6.19,—1.73), FBG (β:—25.26; CI: -53.57,—0.05) and SBP (β:—3.45; CI:—39.62, 0.72) (Table 3).

### Association between stiblen intake (energy-adjusted) and body composition and biochemical parameters

After adjusting for potential confounders, there was a significant negative association between stilbenes intake and BMI (β:—0.34; CI:—0.77,—0.09), HC (β: -0.51; CI: -1.21, 0.18), and CHOL (β:—1.53; CI:—5.38,—2.32), and WC (β:—0.70; CI:—1.76,—0.05) (Table 3).

### Association between other polyphenols intake (energy-adjusted) and body composition and biochemical parameters

After adjusting for potential confounders, there was a significant negative association between other polyphenols intake and TG (β:—0.23; CI:—0.05, 0.41) (Table 3).

### Association between beverages containing total polyphenols (energy-adjusted) and body composition and biochemical parameters

After adjusting for age, physical activity, intake of energy, and supplement, there was a marginal positive association between beverages containing polyphenols and DBP (β: 0.16; CI:—0.001, 0.33) (Supplementary table 3).

### Association between beverages containing flavonoids (energy-adjusted) and body composition and biochemical parameters

After adjusting for potential confounders, there was a marginal positive association between beverages containing flavonoids and HDL-c (β: 0.14; CI:—0.30, 0.08) (Supplementary table 3).

### Association between beverages containing phenolic acids (energy-adjusted) and body composition and biochemical parameters

After adjusting for potential confounders, there was a significant negative association between beverages containing phenolic acids and HC (β:—0.09; CI:—0.36,—0.003) (Supplementary table 3).

## Discussion

The current study investigated associations between DPI and cardiometabolic factors in overweight and obese Iranian women.

The results of this study showed that while there was a  negative association between stilbenes and lignans intake and BMI, stilbenes intake and WC, beverages containing phenolic acids and HC, total polyphenols intake and WHR and WHtR, stilbenes intake and CHOL level, there was a positive association between beverages containing flavonoids intake and HDL-c level. Furthermore, while there was a negative association between other polyphenols intake and TG, lignan intake and FBG, HOMA-IR, and SBP, there was a positive association between beverages containing total polyphenols intake and DBP.

The results of this study showed that higher consumption of lignans and stilbenes was associated with a lower BMI in adult women. While this finding is consistent with Guo et al. 2017 [26], it is inconsistent with Zujko et al. 2018 [15] that found no associations between DPI and BMI. The different results might be due to different sample size which could have an impact on the power of the analysis. While this study included 404 women, Zujko et al. 2018 included 5690 adults [15, 27]. Furthermore, this study found an inverse association between stilbenes intake and WC which was consistent with Guo et al. 2017 study [26].

The results of this study demonstrated that there was a negative association between beverages containing phenolic acids and HC which demonstrated that high phenolic acid intake was associated with lower HC. Furthermore, there was negative associations between total polyphenols intake and WHR, and between total polyphenols intake and WHtR. These findings are in line with previous studies that reported negative associations between total polyphenols intake and WHtR and between abdominal adiposity and stilbenes intake [16, 26, 28]. A possible explanation for the negative association between total polyphenols intake and its subtypes and body composition is that total polyphenols reduces obesity through decreasing TG accumulation, stimulating lipolysis and β-oxidation, inhibition of adipogenesis, and increasing energy expenditure by up-regulating uncoupling protein [29]. As a result, lower intake of polyphenols may be associated with higher anthropometric indices [30, 31]. Consistent with our results, a randomized clinical trial in Japanese participants, showed that intake of capsules containing polyphenols reduced the level of serum CHOL [32]. Furthermore, Nagasako-Akazome et al. 2007 and Castro-Barquero et al. 2020, reported that the higher intake of capsules containing polyphenols and flavonoids was associated with lower LDL-c and higher HDL-c [32, 33].

The results of our study demonstrated that there was a negative association between lignans and HOMA IR and FBG levels. In line with our findings, a study in Polish adults showed a negative link between DPI and its subgroups and risk of type 2 diabetes (32).

The findings of our study showed that the intake of other polyphenols was associated with lower level of TG. This could be explained by the fact that other polyphenols inhibit pancreatic lipase activity which can have an impact on TG levels (33).

In this study, we found a significant association between polyphenol subtypes and blood pressure, which is consistent with Grosso et al. 2018 study that showed a negative association between the subclasses of DPI and risk of high blood pressure [34].

The findings of this study demonstrated an inverse association between DPI intake and metabolic disorders including cardiovascular diseases. It possibly might be attributed to anti-atherogenic properties effects of DPI, including protection of LDL-c from oxidative stress, and prohibition of peroxyl radical-induced DNA sequence failure [35]. Plant bioactive foods, including polyphenols are found in some foods, can play an important role in protecting heart, reducing inflammation and LDL oxidation, and protecting against pathogens. Polyphenols play a significant role in reducing tissue insulin resistance and reducing visceral fat by participating in energy metabolism, altering gene expression (reducing PPAR-γ and increasing uncoupling protein 1). Increasing the activity of an enzymatic protein called adenosine monophosphate-activated protein kinase by regulating glucose and fatty acid transport and increasing fatty acid oxidation and inhibiting and suppressing the gluconeogenesis cycle can lead to improved metabolic factors such as increased insulin sensitivity [36–38].

This study has several strengths. Firstly, this study was the first study that comprehensively evaluated associations between DPI and its components and metabolic outcomes in overweight and obese Iranian women. Secondly, the total polyphenol and its subgroups were measured accurately using Phenol-Explorer.

This study has several limitations. Firstly, this study used the FFQ questionnaire which is dependent on respondent's memory. Secondly, It was not possible to examine the causal association between DPI with cardiometabolic factors due to the cross-sectional study design. Thirdly, this study included only females, thus the results are not generalizable to whole population.

## Conclusion

The present study demonstrated that the intake of polyphenols and their constituents could improve metabolic disorder traits. Given the high prevalence of MetS in the Iranian population, a prospective study that includes a nationally representative sample of Iranian population is needed to be able to understand and compare the current findings.

## Supplementary Information

**Additional file 1: Supplementary table 1. **Foods imported to extract polyphenols. **Supplementary table 2. **Distributionof daily food and nutrient intake across tertiles of energy-adjusted DPI in overweight and obesewomen (*n*= 404). **Supplementary table 3. **Association between beverages containing polyphenols and itssubgroupswith health outcomes in overweight and obese women (*n* = 404).

## Data Availability

Authors declare that the data of this study are provided in this article, and all the data in the study will be available with the opinion of the corresponding author for this study.

## Abbreviations

MetS: Metabolic Syndrome
IDF: International Diabetes Federation
TG: Triglyceride
LDL-c: Low-density lipoprotein cholesterol
HDL-c: High-density lipoprotein cholesterol
DM: Diabetes mellitus
CVD: Cardiovascular disease
BMI: Body mass index
WC: Waist circumference
DPI: Dietary polyphenol intake
FPG: Fasting plasma glucose
IPAQ: International Physical Activity Questionnaire
NC: Neck circumference
HC: Hip circumference
WHR: Waist-to-hip ratio
WHtR: Waist to height ratio
CHOL: Cholesterol
FBG: Fasting bloodglucose
IR: Insulin resistance
HOMA: Homeostatic model assessment
hs-CRP: High-sensitivity C-reactive protein
ELISA: Enzyme-Linked Immunosorbent Assays
SBP: Systolic blood pressure
DBP: Diastolic blood pressure
ANOVA: One-way analysis of variance
SD: Standard deviation
ANCOVA: Analysis of covariance
B: Beta
CI: Confidence interval
