# Supplementary material for: The association between dietary polyphenol intake and cardiometabolic factors in overweight and obese women: a cross-sectional study
Source: BMC Endocr Disord. 2022 May 10;22:120. doi: 10.1186/s12902-022-01025-3 (PMC9088119; doi:10.1186/s12902-022-01025-3)
Supplement: Supplementary file 1 — Additional file 1: Supplementary table 1. Foods imported to extract polyphenols. Supplementary table 2. Distributionof daily food and nutrient intake across tertiles of energy-adjusted DPI in overweight and obesewomen (n= 404). Supplementary table 3. Association between beverages containing polyphenols and itssubgroupswith health outcomes in overweight and obese women (n = 404). [file 12902_2022_1025_MOESM1_ESM.docx]

| **Supplementary table** 1.Foods imported to extract polyphenols | **Fruits and its products** | **beverages** |
| --- | --- | --- |
| **Cereals and cereal products** | Cantaloupe | Tea |
| Barley | Melon | **Oils** |
| grain flour | Watermelon | Olive oil |
| hole grain flour | Pear | Beans |
| [Maize](http://phenol-explorer.eu/foods/402) | Apricot | Chickpea |
| [Rice](http://phenol-explorer.eu/foods/445) | Cherries | Soy |
| wheat | Apple | [Mung bean](http://phenol-explorer.eu/foods/580) |
| **Nuts** | Orange |  |
| Peanut | [Grape](http://phenol-explorer.eu/foods/131) |  |
| Chestnut | Green tomatoes |  |
| Walnut | Fresh figs |  |
| Pistachio | Dried fig |  |
| Hazelnut | Angor |  |
| **Vegetable** | Kiwi |  |
| [Pepper](http://phenol-explorer.eu/foods/705) | Pomegranate |  |
| [Tomato](http://phenol-explorer.eu/foods/387) | Date |  |
| [Zucchini](http://phenol-explorer.eu/foods/502) | Strawberry |  |
| [Onion](http://phenol-explorer.eu/foods/255) | Lemon |  |
| [Green bean](http://phenol-explorer.eu/foods/334) | sour lemon |  |
| [Spinach](http://phenol-explorer.eu/foods/272) | banana |  |
| Carrot | Orange juice |  |
| C[abbage](http://phenol-explorer.eu/foods/283) | Apple juice |  |
| [Broccoli](http://phenol-explorer.eu/foods/1201) | Cantaloupe juice |  |
| Eggplant | Juice |  |

| **Supplementary table** 2:Distribution of daily food and nutrient intake across tertiles of energy-adjusted DPI in overweight and obese women ( n = 404) | | | | | |
| --- | --- | --- | --- | --- | --- |
| **Variables** | **T1** | **T2** | **T3** | **P-value** | **P-value*** |
|  | **Mean**$\boldsymbol{\pm}$**SD** | | |  |  |
| **Food groups** |  | | |  |  |
| **Whole grains (serving/day)** | 4.93±1.15 | 6.10±1.02 | 10.53±0.95^a,b^ | **< 0.001** | **< 0.001** |
| **Refined grains (serving/day)** | 478.93±21.48 | 425.05±19.04 | 414.95±17.78^a^ | **0.05** | 0.08 |
| **Vegetables (serving/day)** | 310.82±26.77 | 403.87±22.73^a^ | 534.04±22.17^a,b^ | **< 0.001** | **< 0.001** |
| **Fruits (serving/day)** | 372.92±31.48 | 558.40±27.90^a^ | 609.62±26.06^b^ | **< 0.001** | **< 0.001** |
| **Legume (serving/day)** | 25.92±3.75 | 39.50±3.32^a^ | 81.82±3.10^a,b^ | **< 0.001** | **< 0.001** |
| **Nuts (serving/day)** | 13.74±1.71 | 14.68±1.52 | 14.26±1.42 | 0.92 | 0.95 |
| **Sugar-sweetened beverages (ml/day)** | 38.50±6.94 | 27.65±6.15 | 13.60±5.74^a^ | **0.02** | **0.03** |
| **Dairy products (serving/day)** | 271.23±24.71 | 288.02±21.90 | 317.05±20.46 | 0.33 | 0.28 |
| **Vegtable oils (serving/day)** | 21.17±2.45 | 24.39±2.17 | 23.95±2.02 | 0.57 | 0.62 |
| **starchy vegetables (serving/day)** | 38.82±3.91 | 40.31±3.46 | 55.00±3.23^a,b^ | **0.001** | **0.001** |
| **Poultry (serving/day)** | 38.40±4.45 | 35.04±3.95 | 34.79±3.69 | 0.79 | 0.74 |
| **Fish (serving/day)** | 11.29±1.39 | 11.19±1.24 | 11.84±1.15 | 0.91 | 0.85 |
| **Red meat (serving/day)** | 20.26±1.96 | 23.51±1.74 | 20.96±1.62 | 0.40 | 0.73 |
| **White meat (serving/day)** | 49.70±5.14 | 46.23±4.56 | 46.64±4.25 | 0.86 | 0.78 |
| **Egg (serving/day)** | 20.69±1.59 | 20.82±1.41 | 23.22±1.31 | 0.34 | 0.27 |
| **Tea (ml/day)** | 875.33±87.88 | 650.45±77.89 | 690.21±72.75 | 0.13 | 0.13 |
| **Coffee (ml/day)** | 22.34±5.80 | 19.66±5.14 | 24.22±4.80 | 0.81 | 0.77 |
| **Fruit juice (ml/day)** | 34.05±4.94 | 29.64±4.38 | 19.86±4.09 | **0.06** | 0.09 |
| **Salt (serving/day)** | 50.72±4.72 | 38.12±4.18 | 39.59±3.91 | 0.10 | 0.09 |
| **Spices (serving/day)** | 2.91±0.21 | 3.11±0.18 | 3.62±0.17^a^ | **0.02** | **0.02** |
| **Energy, macronutrients and miconutreints** |  | | | | |
| **Energy intake (kcal)** | 2672.53±923.40 | 2535.18±780.97 | 2690.62±705.63 | 0.24 | 0.17 |
| **Protein (g/day)** | 86.99±1.88 | 87.09±1.71 | 90.31±1.56 | 0.22 | 0.26 |
| **Carbohydrates (g/day)** | 365.93±5.17 | 365.90±4.70 | 380.68±4.29^b^ | **0.06** | **0.03** |
| **Total fat (g/day)** | 95.55±2.23 | 97.56±2.03 | 90.86±1.85^b^ | 0.66 | **0.04** |
| **CHOL (g/day)** | 255.91±9.76 | 262.04±8.87 | 243.13±8.10 | 0.25 | 0.27 |
| **Trans fat (g/day)** | 0.00±0.00 | 0.00±0.00 | 0.00±0.00 | 0.96 | 0.95 |
| **MUFA (g/day)** | 31.30±0.99 | 32.52±0.90 | 30.23±0.82 | 0.67 | 0.17 |
| **PUFA (g/day)** | 19.26±0.87 | 20.32±0.79 | 20.33±0.72 | 0.57 | 0.58 |
| **EPA (g/day)** | 0.03±0.00 | 0.03±0.00 | 0.03±0.00 | 0.28 | 0.65 |
| **DHA (g/day)** | 0.10±0.01 | 0.10±0.01 | 0.11±0.11 | 0.36 | 0.75 |
| **Oleic (g/day)** | 27.99±1.00 | 29.25±0.90 | 27.21±0.83 | 0.73 | 0.25 |
| **Linoleic acid (g/day)** | 16.69±0.86 | 17.58±0.78 | 17.52±0.71 | 0.69 | 0.69 |
| **Total fiber (g/day)** | 38.03±1.48 | 43.01±1.35^a^ | 51.78±1.23^a,b^ | **< 0.001** | **< 0.001** |
| **Vitamin B1 (mg/day)** | 2.13±0.04 | 2.01±0.03 | 2.09±0.03 | 0.13 | **0.06** |
| **Vitamin B2 (mg/day)** | 2.14±0.06 | 2.20±0.05 | 2.20±0.05 | 0.70 | 0.71 |
| **Vitamin B3 (mg/day)** | 25.65±0.67 | 24.77±0.61 | 25.27±0.56 | 0.31 | 0.62 |
| **Vitamin B6 (mg/day)** | 2.06±0.04 | 2.09±0.04 | 2.27±0.04^a,b^ | **0.01** | **0.001** |
| **Vitamin B9 (mg/day)** | 574.18±10.98 | 572.50±9.98 | 653.53±9.11^a,b^ | **< 0.001** | **< 0.001** |
| **Vitamin B12 (mg/day)** | 4.40±0.23 | 4.67±0.21 | 3.99±0.19^b^ | 0.50 | **0.06** |
| **Vitamin C (mg/day)** | 145.94±11.94 | 207.80±10.84^a^ | 217.62±9.90^a^ | **< 0.001** | **< 0.001** |
| **Vitamin D (mg/day)** | 1.72±0.17 | 2.12±0.15 | 1.98±0.14 | 0.85 | 0.22 |
| **Vitamin E (mg/day)** | 15.54±0.98 | 17.89±0.89 | 18.03±0.81 | 0.12 | 0.10 |
| **Vitamin K (mg/day)** | 160.27±20.78 | 191.22±18.87 | 264.29±17.24^a,b^ | **< 0.001** | **< 0.001** |
| **Retinol (mg/day)** | 648.72±40.21 | 784.32±36.53^a^ | 845.21±33.36^a^ | **0.002** | **0.001** |
| **Sodium (mg/day)** | 4368.80±120.98 | 4161.79±109.90 | 4218.34±100.37 | 0.31 | 0.43 |
| **Potassium (mg/day)** | 3861.65±108.15 | 4214.11±98.25^a^ | 4696.30±89.73^a,b^ | **< 0.001** | **< 0.001** |
| **lycopene (mg/day)** | 5144.99±519.90 | 7236.90±472.32^a^ | 7304.84±431.35^a^ | **< 0.001** | **0.03** |
| **Lutein (mg/day)** | 1616.19±184.02 | 2034±167.18 | 2952.02±152.67^a,b^ | **0.005** | **< 0.001** |
| **Biotin (mg/day)** | 34.50±1.55 | 37.19±1.40 | 41.51±1.28^a^ | **0.003** | **0.002** |
| **Magnesium (mg/day)** | 7.35±0.25 | 6.83±0.23 | 7.01±0.21 | 0.23 | 0.32 |
| **Copper (mg/day)** | 1.85±0.04 | 1.92±0.04 | 2.12±0.04^a,b^ | **0.003** | **< 0.001** |
| **Chromium (mg/day)** | 0.11±0.00 | 0.11±0.00 | 0.10±0.00 | 0.54 | 0.43 |
| **Caffeine (mg/day)** | 175.57±16.83 | 148.01±15.29 | 137.17±13.96 | 0.19 | 0.20 |
| CHOL: cholesterol, MUFA: monounsaturated fatty acid, , PUFA: polyunsaturated fatty acid, EPA: eicosapentaenoic acid, DHA: docosahexaenoic acid  All data are presented as mean±SD  P-value obtained from ANOVA test  P-value * obtained from ANCOVA test for age, physical activity, BMI, total energy intake, and supplement consumption  a: significant compared to tertile 1  b: Significance compared to tertile 2  P-value < 0.05 was considered significant | | | | | |

| **Supplementary table** 3**.** Association between beverages containing polyphenols and its subgroupswith health outcomes in overweight and obese women (n = 404). | | | | | | | | | | | | | | | | | |
| --- | --- | --- | --- | --- | --- | --- | --- | --- | --- | --- | --- | --- | --- | --- | --- | --- | --- |
| **Characteristics** | **Model** | **Total polyphenols (ml/day)** | | | | **Flavonoids (ml/day)** | | | | **Phenolic acids (ml/day)** | | | | **Other polyphenols (ml/day)** | | | |
|  |  | **B** | **CI** | **R^2^** | **P-value** | **B** | **CI** | **R^2^** | **P-value** | **B** | **CI** | **R^2^** | **P-value** | **B** | **CI** | **R^2^** | **P-value** |
| **Body composition** | | | | | | | | | | | | | | | | |  |
| **BMI (kg/m^2^)** | **Crude** | 0.01 | -0.01,0.04 | 0.00 | 0.26 | -0.03 | -0.10,0.03 | 0.000 | 0.29 | -0.02 | -0.06,0.02 | 0.000 | 0.36 | 0.28 | -0.82,1.38 | 0.000 | 0.61 |
|  | **Adjusted** | 0.01 | -0.05,0.07 | 0.25 | 0.72 | -0.02 | -0.17,0.12 | 0.04 | 0.72 | -0.02 | -0.12,0.08 | 0.05 | 0.69 | 0.43 | -1.88,2.74 | 0.04 | 0.71 |
| **WC (cm)** | **Crude** | 0.01 | -0.04,0.07 | 0.00 | 0.63 | -0.03 | -0.19,0.12 | 0.000 | 0.65 | 0.02 | -0.13,0.08 | 0.000 | 0.62 | 0.80 | -1.85,3.46 | 0.002 | 0.55 |
|  | **Adjusted** | 0.01 | -0.13,0.16 | 0.26 | 0.84 | -0.03 | -0.39,0.33 | 0.16 | 0.86 | -0.04 | -0.28,0.20 | 0.06 | 0.73 | 1.42 | -4.17,7.02 | 0.04 | 0.61 |
| **HC (cm)** | **Crude** | -0.01 | -0.06,0.04 | 0.02 | 0.63 | 0.03 | -0.11,0.16 | 0.02 | 0.67 | 0.02 | -0.06,0.12 | 0.12 | 0.53 | -0.91 | -3.24,1.42 | 0.003 | 0.44 |
|  | **Adjusted** | 0.06 | -0.03,0.15 | 0.15 | 0.22 | -0.15 | -3.92,0.08 | 0.25 | 0.20 | -0.09 | -0.36,-0.003 | 0.25 | **0.02** | 1.72 | -1.98,5.42 | 0.05 | 0.36 |
| **NC (cm)** | **Crude** | -0.04 | -0.15,0.06 | 0.007 | 0.41 | 0.10 | -0.16,0.37 | 0.04 | 0.45 | 0.08 | -0.10,0.26 | 0.009 | 0.38 | -2.38 | -6.46,1.88 | 0.007 | 0.27 |
|  | **Adjusted** | -0.02 | -0.15,0.11 | 0.13 | 0.77 | 0.04 | -0.29,0.38 | 0.02 | 0.80 | 0.03 | -0.19,0.26 | 0.03 | 0.76 | -1.44 | -6.63,3.75 | 0.03 | 0.58 |
| **WHR (cm)** | **Crude** | 0.03 | 0.00,0.06 | 0.001 | **0.03** | -0.08 | -0.15,-0.00 | 0.001 | **0.04** | -0.05 | -0.10,-0.00 | 0.02 | **0.03** | 1.22 | -0.08,2.53 | 0.000 | **0.06** |
|  | **Adjusted** | 0.00 | -0.00,0.00 | 0.22 | 0.40 | 0.00 | -0.00,0.00 | 0.22 | 0.34 | 0.00 | -0.00,0.00 | 0.03 | 0.57 | 0.00 | -0.03,0.03 | 0.02 | 0.98 |
| **WHtR (cm)** | **Crude** | 6.77 | 0.00,0.00 | 0.00 | 0.73 | 0.00 | -0.00,0.0 | 0.000 | 0.76 | 0.00 | -0.00,0.00 | 0.000 | 0.69 | 0.00 | -0.01,0.02 | 0.000 | 0.59 |
|  | **Adjusted** | 9.31 | -0.00,0.00 | 0.13 | 0.98 | 2.71 | -0.00,0.00 | 0.13 | 0.98 | 0.00 | -0.00,0.00 | 0.04 | 0.85 | 0.00 | -0.02,0.04 | 0.02 | 0.64 |
| **Biochemical parameters** | | | | | | | | | | | | | | | | |  |
| **FBG (mg/dl)** | **Crude** | 0.02 | -0.04,0.09 | 0.01 | 0.48 | -0.06 | -0.23,0.11 | 0.008 | 0.49 | -0.04 | -0.16,0.07 | 0.01 | 0.43 | 0.55 | -2.78,3.89 | 0.01 | 0.74 |
|  | **Adjusted** | 0.01 | -0.15,0.18 | 0.13 | 0.86 | -0.01 | -0.44,0.40 | 0.13 | 0.93 | -0.05 | -0.34,0.23 | 0.14 | 0.71 | 1.81 | -4.61,8.25 | 0.14 | 0.57 |
| **CHOL (mg/dl)** | **Crude** | 0.10 | -0.16,0.36 | 0.005 | 0.45 | -0.25 | -0.91,0.41 | 0.006 | 0.46 | -0.14 | -0.59,0.30 | 0.002 | 0.52 | 0.45 | -12.14,13.04 | 0.002 | 0.94 |
|  | **Adjusted** | -0.08 | -0.49,0.66 | 0.12 | 0.76 | -0.22 | -1.65,1.21 | 0.12 | 0.76 | -0.14 | -1.10,0.82 | 0.12 | 0.77 | 6.32 | -15.26,27.92 | 0.13 | 0.56 |
| **TG (mg/dl)** | **Crude** | 0.15 | -0.28,0.58 | 0.00 | 0.49 | -0.30 | -1.93,0.77 | 0.001 | 0.57 | -0.34 | -1.07,0.38 | 0.000 | 0.35 | 10.79 | -9.71,31.30 | 0.000 | 0.30 |
|  | **Adjusted** | 0.29 | -0.80,1.38 | 0.22 | 0.59 | -0.65 | -3.36,2.06 | 0.12 | 0.63 | -0.60 | -2.43,1.21 | 0.11 | 0.51 | 20.85 | -19.82,61.53 | 0.12 | 0.31 |
| **HDL-c (mg/dl)** | **Crude** | -0.06 | -0.14,0.14 | 0.004 | 0.10 | 0.14 | -0.05,0.34 | 0.004 | 0.14 | 0.12 | -0.01,0.25 | 0.01 | **0.06** | -3.05 | -6.76,0.66 | 0.000 | 0.10 |
|  | **Adjusted** | -0.06 | -2.39,0.11 | 0.21 | 0.50 | 0.14 | -0.30,0.08 | 0.11 | **0.06** | 0.09 | -0.20,0.39 | 0.21 | 0.53 | -1.18 | -7.86,5.48 | 0.01 | 0.72 |
| **LDL-c (mg/dl)** | **Crude** | -0.05 | -0.23,0.12 | 0.00 | 0.57 | 0.10 | -0.34,0.54 | 0.000 | 0.65 | 0.12 | -0.18,0.42 | 0.000 | 0.43 | -4.49 | -12.96,3.97 | 0.000 | 0.29 |
|  | **Adjusted** | -0.03 | -0.44,0.37 | 0.43 | 0.87 | 0.07 | -0.94,1.08 | 0.13 | 0.89 | 0.07 | -0.60,0.75 | 0.13 | 0.83 | -0.14 | -15.37,15.08 | 0.14 | 0.98 |
| **Insulin (mIU/ ml)** | **Crude** | 0.00 | -0.00,0.00 | 0.00 | 0.61 | 0.00 | -0.00,0.00 | 0.000 | 0.74 | 0.00 | -0.00,0.00 | 0.000 | 0.37 | -0.06 | -0.14,0.01 | 0.001 | 0.13 |
|  | **Adjusted** | 0.00 | -0.00,0.00 | 0.32 | 0.66 | -0.00 | -0.01,0.00 | 0.13 | 0.63 | -0.00 | -0.00,0.00 | 0.13 | 0.74 | -0.00 | -0.16,0.15 | 0.13 | 0.97 |
| **HOMA IR** | **Crude** | 0.00 | -0.00,0.01 | 0.004 | 0.36 | -0.01 | -0.03,0.01 | 0.002 | 0.40 | -0.00 | -0.02,0.00 | 0.01 | 0.27 | 0.16 | -0.28,0.61 | 0.01 | 0.46 |
|  | **Adjusted** | 0.00 | -0.02,0.02 |  | 0.96 | -0.00 | -0.05,0.05 | 0.24 | 0.97 | -0.00 | -0.04,0.03 | 0.16 | 0.90 | -0.01 | -0.87,0.85 | 0.16 | 0.98 |
| **SBP (mmHg)** | **Crude** | 0.05 | -0.04,0.15 | 0.003 | 0.29 | -0.11 | -0.35,0.13 | 0.002 | 0.36 | -0.10 | -0.27,0.05 | 0.01 | 0.20 | 2.76 | -1.90,7.42 | 0.01 | 0.24 |
|  | **Adjusted** | 0.05 | -0.15,0.27 | 0.09 | 0.57 | -0.13 | -0.65,0.39 | 0.20 | 0.62 | -0.10 | -0.46,0.24 | 0.09 | 0.54 | 1.33 | -6.73,9.40 | 0.11 | 0.74 |
| **DBP (mmHg)** | **Crude** | 0.06 | -0.00,0.14 | 0.002 | 0.07 | -0.15 | -0.33,0.03 | 0.004 | 0.10 | -0.13 | -0.25,-0.00 | 0.001 | **0.04** | 2.53 | -0.95,6.02 | 0.01 | 0.15 |
|  | **Adjusted** | 0.16 | -0.001,0.33 | 0.08 | **0.05** | -0.25 | -0.60,0.10 | 0.27 | 0.17 | -0.18 | -0.42,0.05 | 0.05 | 0.13 | 3.14 | -2.39,8.69 | 0.06 | 0.26 |
| BMI: body mass index, WC: waist circumference, HC: hip circumference, NC: neck circumference, WHR: weight to hip ratio, WHtR: weight to height ratio, FBG: fasting blood glucose, CHOL: cholesterol,TG: triglyceride, HDL-c: high-density lipoprotein, LDL-c: low-density lipoprotein, HOMA: homeostasis model assessment, IR: insulin resistance, SBP: systolic blood pressure, DBP: diastolic blood pressure.  All data are presented as B±CI obtained from a linear regression.  P-value for adjustment model: Adjusted for age, physical activity, intake of energy and supplement.  P –value < 0.05 was considered significant.  The analysis was adjusted for energy intake. | | | | | | | | | | | | | | | | | |
